# Debating diversity: a commentary on *Standards of practice in empirical bioethics research*

**DOI:** 10.1186/s12910-018-0306-1

**Published:** 2018-07-12

**Authors:** Stacy M. Carter

**Affiliations:** Research for Social Change, Faculty of Social Science, Building 233, G05, Innovation Campus, The University of Wollongong, Wollongong, 2522 Australia

**Keywords:** Methodology for empirical bioethics, Qualitative research

## Abstract

This article provides a commentary on *Standards of practice in empirical bioethics research* by Ives and colleagues (in this Issue). There is much to admire in the paper, and in the demanding consensus-building process on which it reports. I discuss the problems and limits of methodological standardisation, and a central conceptual tension that appears to have divided participants. I suggest that the finished product should be understood as a record of a methodological conversation, rather than being used as a disciplinary tool to limit the evolution of empirical bioethics.

Ives and colleagues’ *Standards of practice in Empirical Bioethics research: Towards a consensus* [1] documents a consensus-building process that hinged on a meeting at Fondation Brocher in 2015. The authorship team deliberated during and following that meeting over what standards for empirical bioethics research they could collectively endorse.

This result is a real achievement, and I agree with much of it: for example, with the aims under Domain Four, which advocate quality and reflexivity in empirical methods. Ha! (I hear anyone who knows me shout) but of course you agree with those. You’re an empirical researcher.

This points to a central problem in methodological pronouncements: the positioning of the pronouncer/s. I am an empirical researcher, so I defend robust empirical methodologies. In contrast, I have colleagues from a philosophical background who make little epistemic distinction between a systematic empirical research project and having a chat with a few friendly policymakers; they see either as mere background for making a philosophical argument, and are much more concerned about practice standards in this domain.

This is unsurprising: it is well-recognised that knowledge, and standards for knowledge production, are inevitably situated [2]. So it is to the authors’ credit that they show their workings, including the disciplinary locations of the 16 scholars involved, the effort required, and the disharmony that sometimes sat under apparent consensus. I do have concerns, discussed below, but many of these have been anticipated by the authors, who are cautious in their claim-making. My comments should be seen not as criticism but as a discussion of the implications of such exercises.

## On standardisation

The attempt to nail down methodological standards is as old as research textbooks and rife in the biomedical literature [3]. The authors suggest similar work is needed for empirical bioethics, promising a range of potential benefits. Better-established sub-disciplines, they propose, share widely-accepted conventions. They can thus communicate more efficiently (less explanation is needed), their funders, editors and reviewers can ensure ‘minimum methodological quality’ [1], and they can more easily build ‘communities of practice’ [1], including through teaching.

Prima facie this seems fair enough, but in practice it is often not so simple, partly because of a problem with the level of abstraction. There have now been decades of debate, for example, about quality standards for ‘qualitative research’ [4, 5]. The broad church of qualitative inquiry contains diverse practices, disciplinary roots and methodological and epistemic commitments. As you move from the general to the particular (e.g. from ‘qualitative research’ to ‘Charmaz’s iteration of Grounded Theory’ [6]) it becomes easier to agree on quality criteria. Although general criteria—including widely-used checklists—exist, [7] they mask enormous diversity and disagreement.

The same problem, unsurprisingly, occurred in this project: high consensus, expressed as percent agreement scores, sometimes disguised heated debate, and standardisation was sometimes impossible (e.g. on what constitutes ‘a basic understanding of bioethics’ in Aim 14). But this is not a failure: it is one more example of a common methodological pattern.

At this point, however, a caution. Standardisation might be difficult and in the realm of the ideal. But once expressed, standards can easily be used as concrete boundary-drawing tools, especially by inexperienced or inexpert scholars who have a functional need for a rulebook (students, newcomers, editors working across fields). Summary lists or tables are especially prone to appropriation. These authors have tried hard to resist this by providing context and detail; readers should reciprocate by being careful in how they use this work.

## What is empirical bioethics?

As I finished drafting this commentary, I noticed footnote d, which acknowledges tension between Aim Two and Aim 12. That footnote d exists is further evidence of the authors’ commitment to plain dealing with the reader. It also gestures at a deeper fracturing in the process that was already troubling me, with implications for many of the resulting domains and aims. If I have interpreted correctly, participants divided along a central fissure in empirical bioethics [8–10]. Some assumed that the descriptive and the normative were separable and so required active integration; others thought they were inevitably entangled. I am in the latter camp, and so see through that lens, perhaps reflecting long empirical experience with the complexity and always-morally-charged nature of everyday sense-making [11].

This tension is in part a problem of circularity. Prior to deliberation participants were asked to accept a conception of empirical bioethics as ‘integrated’, which seems to smuggle in the assumption of separability. This assumption seems to dominate in Aim Two, which formalises strong limitations on empirical bioethics (albeit with low agreement): only deductively-designed research that adjudicates between two courses of action or two versions of a concept should count; research on ‘the form and nature of how ethical issues arise in practical situations’ [1] is explicitly excluded. This, it seems to me, slices off much useful work from Australia, the UK and elsewhere that explains the moral reasoning and practices of lay people and professionals.

As the process developed however (and again, if my interpretation is correct), there seems to have been an uprising. At Aim Six, despite 100% agreement about explaining methods for integration, someone has inserted a caveat that a meta-ethical/epistemological position could be set out that ‘either makes integration unnecessary or provides an alternative account of the relationship between facts and values’ [1]. At Aim 12 [1], after long debate and despite strong disagreement, it was admitted that developing ‘new insights that could broaden one’s moral horizon’, or explanations of ‘relevant aspects of the problem’ should count as ‘normative analysis’ [1].

It’s important that we notice this tension. It suggests we should not take the agreement scores in this paper as prescriptions regarding *how we should do empirical bioethics*. Perhaps instead, we could read this as a meticulous record of ongoing disagreements over empirical bioethics, explained in part by the positioning of participants.

## Conclusions

If this reading is accepted, what should be done with the resulting document? The authors themselves claim that the standards are largely ‘formal rather than contentful’ [1], so do not stipulate how researchers should act (implying, I think, that they can be applied without doing too much damage). But if formal standards smuggle in contentful assumptions about what constitutes empirical bioethics, or mask real disagreement, this distinction may not stand.

The authors acknowledge that the results cannot be a universal or binding statement, only a movement “*towards* a consensus” [1]. Inevitably, we cannot know how generalisable or replicable this process is without running similar processes in other contexts (replicability is, somewhat ironically, an empirical question). Perhaps, as has been the case in qualitative inquiry, empirical bioethics needs to go through a period of debating its own diversity (which these authors understand better than most [12]) so as to understand what different approaches can contribute to the whole. Qualitative inquiry has survived and thrived as a diverse set of complementary practices for many decades, despite periodic attempts at restriction. Empirical bioethics may follow a similar path. In the meantime, based on the history of methodological standards elsewhere, it seems wise to treat such exercises as the continuation of a conversation, rather than as a tool to separate the methodological sheep from the metaphorical goats.
